# Comparative analysis of the correlation between anxiety, salivary alpha amylase, cortisol levels, and athletes’ performance in archery competitions

**DOI:** 10.20463/jenb.2018.0032

**Published:** 2018-12-31

**Authors:** In-Soo Lim

**Affiliations:** 1 Department of physical education, Changwon National University, Gyeongsan Korea

**Keywords:** anxiety, salivary alpha amylase, salivary cortisol, and archery competitions

## Abstract

**[Purpose]:**

This study aimed to analyze the correlation between anxiety, salivary alpha amylase (sAA), and salivary cortisol (sC) changes in athletes showing a high or low level performance in an actual archery competition.

**[Methods]:**

The participants were female university athletes who participated in the individual 50 m archery competition at the 21st Korean National Archery Team Trials, in July 2018. Based on their game record in the competition, 9 athletes were allocated to the high-performance group (HPG) and another 9 to the low-performance group (LPG). Anxiety caused by the competition was rated on a 1–10 point Likert scale at 30 min before competition (pre-30), 3 min before competition (pre-3), and 30 min after competition (post-30). This assessment method directly measured their cognitive anxiety. Saliva samples were collected in a tube by having the athletes chew on an absorber swab. For data analysis, two-way ANOVA with repeated measures was performed and Pearson’s correlation method was applied to correlate the variables.

**[Results]:**

In the actual competition, significant difference between the game records of the HPG (mean score 339.5±4.1) and the LPG (mean score 323.3±3.4) was observed. Competitive anxiety showed a significant decrease in the HPG compared to the LPG. Due to the competition, sAA and sC were significantly decreased in the HPG compared to the LPG. Analysis of correlations between competition scores, anxiety, sAA, and sC, revealed that lower anxiety was associated with higher scores in the HPG. Pre-3 anxiety positively correlated with pre-3 sAA and sC. In the LPG, lower scores were associated with persistent anxiety until the completion of the competition. Positive correlations were observed for pre-3 anxiety with post-30 sC, pre-3 sAA with post-30 sAA and sC, and pre-3 cortisol with post-30 sAA.

**[Conclusion]:**

Increased anxiety in the actual archery competition was associated with significant increase in sAA and sC. Elevated sAA and sC from prior-competition to post-competition stage were associated with reduced performance. Significant correlations between the measured variables (game records, anxiety, sAA, and sC) were associated with worse performance.

## INTRODUCTION

Archery was selected as an official event for the 1900 Paris Olympic Games, but was subsequently omitted, and only included again as an event in the 1972 Munich Olympic Games. In Korea, archery has been broadly considered as a school sport since 1960s and 70s, and Korea has maintained its status as a world leader in the sport until today. Since psychological factors have a decisive influence on an archer’s performance, archery, even more than other sports, is described as a mental sport. In actual competitions, victory and defeat are often affected more by the athletes’ anxiety, tension, stress, and pressure than by their technical skill.

 In general, stress activates the hypothalamic pituitary adrenal (HPA) axis and the sympathetic adrenal medullary (SAM) system. Activation of the HPA axis increases cortisol, and activation of the SAM system increases catecholamines^1,2^. Studies evaluating stress often analyze blood cortisol and catecholamine levels. Since blood collection itself can cause stress, there are difficulties in collecting blood from athletes participating in a sports competition. Recently, field studies have employed noninvasive measurements using saliva, rather than blood^3,4^. In particular, while salivary cortisol (sC) significantly reflects changes in blood cortisol levels, blood catecholamine levels are better represented by salivary alpha-amylase (sAA) than by salivary catecholamines^5^. sAA is elevated by sympathetic nervous activity in physically or psychologically stressful situations, and shows a faster response than sC^6-8^.

 The competitive anxiety that athletes experience in most of the sports competitions is known to be associated with elevated sAA and sC^9-11^. For example, in athletes participating in an inline skating competition, sAA and sC were significantly increased before the competition compared to resting condition^12^. In golf and soccer, sAA and sC increased significantly in actual match compared to practice matches^13^. In official taekwondo competition, sAA and sC showed a significant increase until just after a bout, and then showed a significant decrease 30 min after the bout^14^. In athletes participating in tennis matches, the increase in competitive anxiety was directly correlated with an increase in sC^15^. Thus, in athletes participating in sports competitions, changes in sAA and sC can be used as biomarkers to evaluate anxiety^16,17,18^. However, there is still a lack of knowledge for sAA and sC to be considered as physiological and psychological anxiety markers in athletes showing different levels of performance in actual competition. In particular, where most previous studies have focused on ball sports, we could not find any study on archery, even though it is a mental sport, which is very sensitive to psychological factors. Therefore, in this study, we analyzed the correlations between competitive anxiety, sAA, and sC in high-performing as well as low-performing athletes in an actual archery competition.

## METHODS

### Subjects

The subjects consisted of 24 female university athletes who participated in the individual 50 m archery competition at the 21st Korean National Archery Team Trials, taking place between 2:00 pm and 4:00 pm on July 14th, 2018. Based on their game records in the competition, 9 athletes were allocated into the high-performance group (HPG) and another 9 athletes were allocated into the low performance group (LPG). The HPG ranked between 1st and 10th place, and all scored at least 330 points, while the LPG ranked between 20th and 30th place, and all scored 329 points or lower. The subjects consisted of individuals with at least 10 years of archery experience, no current medications or clinical disease, and especially no intraoral disease or inflammation. In the HPG, the mean age was 21.6±1.8 years, the mean height was 164.3±3.8 cm, and the mean body weight was 53.5±3.6 kg. In the LPG, the mean age was 21.2±1.5 years, the mean height was 167.9±3.2 cm, and the mean body weight was 55.0±5.3 kg.

### Assessment of anxiety and game record

Competitive anxiety caused by the archery competition was assessed on a Likert scale from 1 point being the lowest (“Not at all anxious”) to 10 points being the highest (“Very anxious”), based on the scale of Beck et al. [19]. This method was used to assess cognitive anxiety 30 min prior the competition (pre-30), 3 min prior the competition (pre-3), and 30 min after the competition (post-30). Game records were evaluated based on the scores. There were 6 rounds of 50 m competition, each consists of 6 shots, and therefore, the scores were the sum of scores from total 36 shots.

### Saliva collection and analysis

All the athletes were provided with same lunch between 11:00 am and 12:00 am, after which they were forbidden from brushing their teeth or having snacks, including coffee. The competition began at 2:00 pm, and saliva samples were collected at 30 min before the competition, 3 min before the competition, and 30 min after the competition. Subjects were allowed to wash their mouths with water 10 minutes before the saliva sample collection. After chewing on a cotton swab 20 times, saliva was gathered in a tube and stored frozen. For sAA and sC analysis, we used a VERSA Max Microplate Reader (Molecular Devices, USA) to perform ELISA (enzyme-linked immunosorbent assay). The reagents were sAA and sC assay kits (Salimetrics).

### Statistical analysis 

SPSS 23.0 was used for statistical analysis. Means (M) and standard deviations (SD) were calculated for the measured variables, and differences in the means were tested using a two-way ANOVA with Repeated Measures. In the case of an interaction effect, one-way ANOVA was used to analyze differences between different time points within each group, and independent t-tests were used to analyze differences between the groups at each time point. In addition, correlations between the measured variables in each group were analyzed using Pearson’s correlation method. All statistical tests were performed at a significance level (α) of 0.05.

## RESULTS

### Comparison of game record between HPG and LPG 

In the actual 50 m individual archery competition, the mean score for the HPG was 339.5±4.1 (individual scores: 347, 345, 341, 340, 338, 338, 337, 335, and 335), and the mean score for the LPG was 323.3±3.4 (individual scores: 328, 327, 327, 324, 324, 321, 320, 320, and 319). There was a significant difference between the scores of the HPG and those of the LPG (t=8.95, p<0.001).

### Comparison of anxiety between HPG and LPG 

The anxiety scores of the HPG at 30 min before, 3 min before, and 30 min after the competition were, 3.33±0.8, 4.88±10, and 2.74±0.6, respectively. The anxiety scores of the LPG at 30 min before, 3 min before, and 30 min after the competition were 4.66±1.2, 6.14±1.5, and 4.52±1.3, respectively. There was a significant difference between the HPG and LPG in anxiety over time (F=97.1, p<0.001), and post-hoc tests showed significant differences between the groups at all three time points (p<0.05).

### Comparison of sAA levels between HPG and LPG

The sAA levels of the HPG at 30 min before, 3 min before, and 30 min after the competition were 28.1±6.2 U/mL, 51.6±15.8 U/mL, and 42.3±10.5 U/mL, respectively. The sAA levels of the LPG at 30 min before, 3 min before, and 30 min after the competition were 31.0±7.1 U/mL, 67.4±9.7 U/mL, and 60.1±13.7 U/mL, respectively. These results showed a statistically significant difference between the two groups (F=87.5, p<.001). Post-hoc testing showed that there was no significant difference at 30 min before the competition, but that sAA levels were significantly different between the two groups at 3 min before and 30 min after the competition (p<0.05). 

### Comparison of sC levels between HPG and LPG

The sC levels of the HPG at 30 min before, 3 min before, and 30 min after the competition were 1.23±0.2 µg/dL, 1.26±0.1 µg/dL, and 1.46±0.2 µg/dL, respectively. The sC levels of the LPG at 30 min before, 3 min before, and 30 min after the competition were 1.22±0.7 µg/dL, 1.50±0.1 µg/dL, and 1.98±0.2 µg/dL, respectively. Upon comparison between groups, there was a significant difference in sC over time (F=74.2, P<.001). Post-hoc tests showed no significant differences between the HPG and LPG at 30 min before and 3 min before the competition, but it did show statistically significant difference (p<.05) in sC at 30 min after the competition.

**Figure 1.**
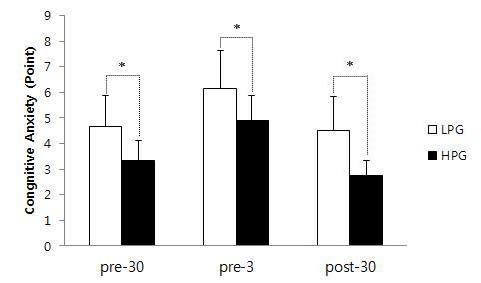
Comparison of the cognitive anxiety level during pre-30, pre-3, and post-30 between LPG and HPG. sC : salivary cortisol; LPG : low performance group; HPG : high performance group; pre-30 : 30 min before competition; pre-3 : 3 min before competition; post-30 : 30 min after competition; *: p<0.05

**Figure 2.**
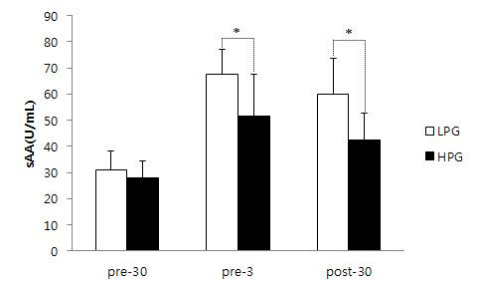
Comparison of the sAA level during pre-30, pre-3, and post-30 between LPG and HPG. sAA: salivary alpha amylase; LPG : low performance group; HPG : high performance group; pre-30 : 30 min before competition; pre-3 : 3 min before competition; post-30 : 30 min after competition; *: p<0.05

**Figure 3.**
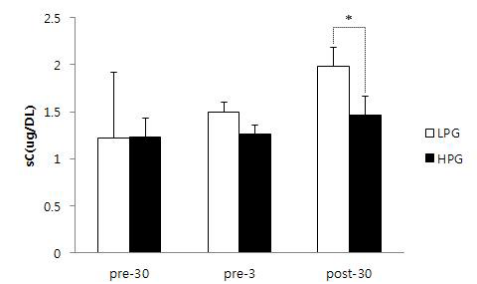
Comparison of the sC level during pre-30, pre-3, and post-30 between LPG and HPG. sC : salivary cortisol; LPG : low performance group; HPG : high performance group; pre-30 : 30 min before competition; pre-3 : 3 min before competition; post-30 : 30 min after competition; *: p<0.05

### Correlations between game record, anxiety, sAA, and sC

Table-1 shows the results from the analysis for correlations between game records, anxiety, sAA, and sC in the HPG. There was a negative correlation between game record (REC) and anxiety at 3 min before the competition (ANX2), meaning that lower anxiety was associated with better performance. ANX2 also showed positive correlations with sAA and cortisol at 3 min before the competition (AMY2, COR2). 

**Table 1.** Correlation between game record, anxiety, sAA, and cortisol in HPG.

	REC	ANX1	ANX2	ANX3	AMY1	AMY2	AMY3	COR1	COR2	COR3
REC										
ANX1										
ANX2	-.86**									
ANX3										
AMY1										
AMY2			.76*							
AMY3										
COR1										
COR2			.67*							
COR3										

sAA (salivary alpha amylase); sC (salivary cortisol); HPG (high performance group); REC (game record)

ANX1 (anxiety 30 min before competition); ANX2 (anxiety 3 min before competition); ANX3 (anxiety 30 min after competition); AMY1 (sAA 30 min before competition); AMY2 (sAA 3 min before competition); AMY3 (sAA 30 min after competition); COR1 (sC 30 min before competition); COR2 (sC 3 min before competition); COR3 (sC 30 min after competition); *p<0.05; **p<.001

Table-2 shows the results from analysis for correlations between game record, anxiety, sAA, and sC in the LPG. Game record (REC) showed a negative correlation with anxiety at 30 min after the competition (ANX3), meaning that worse performance was associated with anxiety persisting until after the end of the competition. In addition, there was a positive correlation between anxiety at 3 min before the competition (ANX1) and sC at 30 min after the competition (COR3). sAA at 3 min before the competition (AMY2) showed positive correlations with sAA and sC at 30 min after the competition (AMY3 and COR3). In addition, sC at 3 min before the competition (COR2) showed a positive correlation with sAA at 30 min after the competition (AMY3).

**Table 2.** Correlation between game record, anxiety, sAA, and sC in LPG.

	REC	ANX1	ANX2	ANX3	AMY1	AMY2	AMY3	COR1	COR2	COR3
REC										
ANX1										
ANX2		.88**								
ANX3	.72*	.89**	.67*							
AMY1										
AMY2										
AMY3						.73*				
COR1										
COR2							.75*			
COR3			.75*			.69*				

sAA (salivary alpha amylase); sC (salivary cortisol); LPG (low performance group); REC (game record)

ANX1 (anxiety 30 min before competition); ANX2 (anxiety 3 min before competition); ANX3 (anxiety 30 min before competition); AMY1 (sAA 30 min before competition); AMY2 (sAA 3 min before competition); AMY3 (sAA 30 min after competition); COR1 (sC 30 min before competition); COR2 (sC 3 min before competition); COR3 (sC 30 min after competition); *p<.05; **p<.01

## DISCUSSION

In the actual archery competition, the participants in HPG were ranked 1st to 10th and had a mean score of 339.5±4.1, while the participants in LPG were ranked 20th to 30th and had a mean score of 323.3±3.4. Cognitive anxiety during the competition was higher in the LPG than the HPG, and remained higher even after the end of the competition. In the HPG, game record (REC) was negatively correlated with anxiety at 3 min before the competition (ANX2), meaning that lower anxiety was associated with better performance. In the LPG, game record (REC) was negatively correlated with anxiety at 30 min after the competition (ANX3), meaning that athletes with worse performance maintained higher levels of anxiety even after the end of the competition. In a previous study that analyzed cognitive anxiety in 106 athletes, there was a strong negative correlation between athletic performance and anxiety^20^. In tennis trials, compared to losers, winners showed significantly lower cognitive anxiety and significantly higher self-confidence before the match^21^. In our study, in interviews with the athletes after the competition had ended, lower ranked athletes showed persistent emotions of regret, self-blame, and difficulty accepting the results. This psychological state is likely to act as a negative factor in subsequent competitions.

In our study, sAA showed a significant increase from 30 min before the competition to 3 min before the competition, and then showed a trend for a decrease at 30 min after the competition. sAA showed a larger increase in the LPG than the HPG, demonstrating a higher level of both anxiety and physical stress. Meanwhile, sC increased from 30 min before the competition (COR1) to 3 min before the competition (COR2), and showed a trend for an increase until 30 min after the competition (COR3). These patterns are consistent with those reported in previous studies for other types of sport^11^. Specifically, in an official taekwondo competition, sAA increased significantly until immediately after the competition, and then decreased by 30 min after the competition; sC remained elevated up to 30 min after the competition, and decreased thereafter. In our study, sC showed a significant increase at 30 min after the competition in the LPG compared to the HPG, demonstrating that anxiety remained higher even after the competition had ended. When we analyzed correlations between game record, anxiety, sAA, and sC, the HPG showed a negative correlation between game record (REC) and anxiety at 3 min before the competition (ANX2), meaning that lower anxiety was associated with better performance. In addition, anxiety at 3 min before the competition (ANX2) was positively correlated with sAA and sC at 3 min before the competition (AMY2 and COR2). Thus, higher anxiety immediately before the competition was associated with elevated sAA and sC. When the correlation between anxiety and sC was analyzed, there were no significant correlations in the HPG. However, in the LPG, anxiety at 3 min before the competition (ANX2) was positively correlated with sC at 30 min after the competition (COR3). In other words, in the LPG, the higher level of anxiety immediately before the competition was associated with increased sC after the competition had finished. Under stressful conditions, sAA is known to be more sensitive and to response faster than sC^18^. In the LPG, game record (REC) showed a negative correlation with anxiety at 30 min after the competition (ANX3), meaning that worse performance was associated with anxiety persisting until after the end of the competition. In addition, anxiety at 3 min before the competition (ANX1) was positively correlated with sC at 30 min after the competition (COR3), indicating that the increase in sC after the end of the competition is related to the level of anxiety before the competition. In particular, sAA at 3 min before the competition (AMY2) showed positive correlations with sAA and sC at 30 min after the competition (AMY3 and COR3), indicating that the increase in sAA immediately before the competition influenced the elevated sAA and sC after the end of the competition. Moreover, there was a positive correlation between sC at 3 min before the competition (COR2) and sAA at 30 min after the competition (AMY3), demonstrating that increased sC immediately before the competition influenced the elevated sAA after the competition. A previous study reported that, compared to high-performing basketball players, low-performing basketball players showed significant positive correlations between anxiety, sAA, and sC^22^. Another study observed significantly higher anxiety and sC on the first day of competition at a tennis tournament, and reported that losing athletes showed significantly higher anxiety and sC compared to those in the winning athletes^23^.

It is proposed that competitive anxiety increases sAA and sC via interactions between the HPA axis and the SAM system. In particular, elevated sAA and sC after the competition in low-performing athletes compared to high-performing athletes could be due to delayed homeostasis in the biofeedback processes of the HPA axis and SAM system. While there were only 3 significant correlations between the measured variables in the HPG, the LPG showed 8 significant correlations. These results suggest that there may be more correlations between psychological anxiety and physiological anxiety factors that could lead to reduced athletic performance. In conclusion, competitive anxiety in archery increases sAA and sC levels, and we propose elevated sAA and sC from immediately before the competition until after the end of the competition as a factor in reduced performance.
